# A novel combinatorial therapeutic strategy using resveratrol and viral oncolysis for diffuse intrinsic pontine glioma: an in vitro study

**DOI:** 10.1007/s13205-026-05029-x

**Published:** 2026-08-25

**Authors:** Karthik Gourishetti, Isaiah Davis, Shiv Patel, Karthik Rangavajhula, Christian Jacobsen, Monique Ruschel Kanitz, Darshini Kuruppu, Deepak Bhere

**Affiliations:** 1 Biotherapeutics Laboratory, University of South Carolina Floyd School of Medicine, Columbia, SC USA; 2 Department of Pathology, Microbiology and Immunology, University of South Carolina Floyd School of Medicine, Columbia, SC USA; 3 Division of Gastrointestinal and Oncologic Surgery, Massachusetts General Hospital, Harvard Medical School, Boston, MA USA

**Keywords:** Brain cancer, Pediatric brain cancer, Pediatric oncology, Oncolytic virus, Novel delivery strategies

## Abstract

Pediatric central nervous system (CNS) tumors, specifically Diffuse Midline Gliomas (DMG) are a leading cause of cancer-related deaths in children presenting an unmet need for novel therapeutic development. In this study, we assessed the combined effects of resveratrol (RSV) and the oncolytic herpes simplex virus (oHSV), hrR3 in patient-derived diffuse intrinsic pontine glioma (DIPG) cell lines. RSV or hrR3 monotherapy resulted in a dose- and time-dependent reduction in cell viability. RSV pretreatment followed by hrR3 infection led to a significantly greater reduction in tumor cell viability than monotherapy. RSV pretreatment further enhanced the replication of hrR3, as evidenced by increased viral titers. Mechanistic analysis showed that the combination therapy was associated with reduced phosphorylation of the signal transducer and activator of transcription 3 (STAT3) and protein kinase B (AKT) signaling pathways, along with increased expression of apoptotic markers, including cleaved caspase-3 and Poly (ADP-ribose) polymerase (PARP). Together these findings indicate that RSV potentiates hrR3-mediated oncolysis in DIPG cells by enhancing viral replication, suppressing survival signaling and promoting apoptosis in DIPG cells in vitro.

**Supplementary Information:**

The online version contains supplementary material available at https://doi.org/10.1007/s13205-026-05029-x.

## Introduction

Pediatric central nervous system (CNS) tumors represent the leading cause of cancer-related mortality in children, with Diffuse Midline Gliomas (DMG), specifically those arising in the pons; formerly termed Diffuse Intrinsic Pontine Glioma (DIPG) constituting the most lethal subset (Price et al. 2024). While adult high-grade gliomas (HGG), such as Glioblastoma (GBM), often present as contrast-enhancing supratentorial masses with significant neoangiogenesis, DIPG is anatomically positioned within the brainstem (Buczkowicz et al. 2014). This critical region of the brain controls essential autonomic functions, precludes surgical resection, thereby eliminating the primary therapeutic pillar utilized in adult HGG (Theeler et al. 2015). Pediatric CNS tumors are the leading cause of cancer-related mortality among children. Epidemiological data from the Central Brain Tumor Registry of the United States (CBTRUS), indicate these tumors account for around 25% of all childhood cancers, with an incidence rate of approximately 6.05 cases per 100,000 children each year (Price et al. 2025).

The clinical management of DIPG has remained unchanged for decades (Grasso et al. 2015). Focal radiotherapy (RT) has been the main intervention that provides transient clinical benefits, typically extending survival by approximately 3 months (Lo Greco et al. 2023). Despite various attempts to intensify treatment through hypofractionation RT or the addition of cytotoxic radiosensitizers, no systemic agent has successfully improved outcomes over RT alone (Veldhuijzen van Zanten et al. 2015). This lack of progress is primarily due to the blood-brain barrier (BBB) in the pontine region, which severely limits the penetration of standard chemotherapeutics (Bredlau and Korones 2014).

Oncolytic virotherapy has emerged as a compelling alternative to traditional small-molecule therapies (Kaufman et al. 2015). Unlike conventional drugs, oncolytic viruses (OVs) have the preferential ability to target tumor cells and impart antitumor immunity by stimulating the immune system without adverse effects to normal cells (Li et al. 2022; Kan et al. 2020). The OV genome offers engineering potential to target specific cells or armed to act as replication-deficient viral vectors for therapeutic genes (Hamad et al. 2023; Nguyen and Saha 2021). oHSV is a compelling subclass of OVs that possess a highly stable genome, potent cytolytic capability, versatility with genomic engineering, and has availability of anti-herpetic drugs to treat potential adverse reactions (Nguyen and Saha 2021; Totsch et al. 2024). Previous research has shown anti-tumor efficacy of unarmed, rearmed, and armed OV monotherapies to be limited, with more recent oHSV research shifting to combinational multimodal approaches (Nguyen and Saha 2021). Notably, hrR3 an oHSV mutant, exhibits selective replication in tumors and can serve as a platform for delivering therapeutic agents (Kulu et al. 2009). The hrR3 mutation involves the LacZ gene, which encodes β-galactosidase, and a modification of the large subunit of ribonucleotide reductase (RR) in herpes simplexvirus (HSV), enhancing its tumor-targeting capabilities (Sokolowski et al. 2015).

One potential candidate for combinational therapies is RSV, a polyphenolic compound found in grapes, soy, various berries, and peanuts (Cilibrasi et al. 2017; Zhang et al. 2023). RSV has been extensively studied in cancer treatment for its noteworthy pro-apoptotic, antioxidant, anti-inflammatory, and anti-proliferative functions (Zhang et al. 2023). RSV elicits its effects through regulation of cell proliferation, cell cycle progression, reactive oxygen species, apoptotic pathways, and autophagy (Zhang et al. 2023; Cilibrasi et al. 2017). This includes downregulation of the Wnt signaling pathways, which are known to be involved in glioma stem cell maintenance and drug resistance (Cilibrasi et al. 2017). RSV can easily pass through the BBB presenting promise in glioma therapies by alleviating tumor-mediated intracranial pressure (Kim and Song 2021).

In this study, we examined the potentiation of hrR3 viral oncolysis through pretreatment with RSV in DMG/DIPG cells. Based upon growing evidence of combinational therapies having enhanced efficacy in comparison to monotherapies, we evaluated this combination approach to target DMG/DIPG.

## Materials and methods

### Cell lines and culture conditions

DMG/DIPG cells PBT-29FHTC and PBT-24FHTC were sourced from the brain tumor resource lab at Seattle Children’s Hospital via a Material Transfer Agreement. These cells were cultured in NeuroCult Complete media, comprising NeuroCult Neurosphere-Adherent (NS-A) Basal Medium with NS-A Proliferation Supplement (NeuroCult NS-A Proliferation Kit, #05751, STEMCELL Technologies, Cambridge, MA), 1% penicillin-streptomycin (Thermo Fisher Scientific, USA), 20ng/mL Human recombinant fibroblast growth factor-2, and 20ng/mL (#78003 STEMCELL Technologies, Cambridge, MA) Human recombinant epidermal growth factor (#78006 STEMCELL Technologies, Cambridge, MA). The plates, dishes, and flasks were coated with Laminin (Laminin, Mouse, 1 mg, #354232, Corning^®^ (Corning, NY) at a 5–10 µg/cm² concentration. Vero Cells obtained from the American Type Culture Collection (ATCC) (Manassas, VA) were cultured in Dulbecco’s Modified Eagle Medium (DMEM) with 10% heat inactivated fetal bovine serum (HI-FBS) (Gibco #A52568, Thermo Fisher Scientific, USA) with 1% antibiotic solution (100 µg/mL streptomycin, 100 U/ mL penicillin). Both cell lines were maintained in an environment with 5% CO_2_ at 37 °C. Resveratrol was procured from Sigma Aldrich (St. Louis, MO) and 100 mM stocks were made in cell culture grade dimethyl sulfoxide (DMSO). RSV stock solution (100 mM in DMSO) was diluted to working concentrations, and the final DMSO concentration was maintained at ≤ 0.15% across all experimental and control conditions.

### Bioinformatics analysis

Clinical data on survival outcomes for DIPG patients were accessed through the PedcBioPortal for Childhood Cancer Genomics (PedcBioPortal). We queried the Pediatric Brain Tumor Atlas dataset and Kaplan-Meier survival curves were generated to compare overall survival (OS) between different treatment modalities. Statistical significance was assessed using the log-rank test.

### Amplification, purification and titration of hrR3

hrR3 was amplified in Vero cells using a low multiplicity of infection (MOI) and then purified following previously established protocols. In brief, Vero cells were seeded in a T-150 cm^2^ flask at a volume of 3–5 × 10^6^ cells/flask. After 24 h, the cells were infected with hrR3 at 0.01 MOI. At 72 h post-infection (hpi), cells were collected and subjected to freeze-thawing and centrifugation at 2000 revolutions per minute (RPM) for 10 min at 4 °C. The resulting supernatant was filtered through a 0.45 μm filter, and then carefully layered on top of a 25% sucrose solution and centrifuged at the specified RPM for 2 hours at 4 °C. The resulting pellet was resuspended in 10% glycerol in phosphate-buffered saline (PBS), incubated on ice for 10–20 min, and stored as 10 µL aliquots at -80 °C. The titration of hrR3 was performed by X-gal (5-bromo-4-chloro-3-indolyl β-D-galactopyranoside) staining as previously described (Nguyen et al. 2021). hrR3 was a kind gift from our collaborator, Darshini Kuruppu.

### Cell viability assay

Cells were seeded at a density of 5 × 10^3^ cells per well in 96-well plates. Following a 24-hour incubation, the cells were pre-treated with varying concentrations of RSV. After 24 h of RSV pre-treatment, the cells were infected with different MOIs of hrR3. Cell viability was assessed using the sulforhodamine B (SRB) assay as per the previously referenced method (Vichai and Kirtikara 2006). Cell viability was calculated using the following formula for each treatment: Cell viability (%) = (mean absorbance in infected cells / mean absorbance in control (untreated) cells) × 100.

### Viral replication assay

A viral replication assay was performed as previously described (Hu et al. 2021). Briefly, PBT29FHTC cells were incubated with RSV for 24 h and then infected with hrR3 for 1 h. Supernatants were harvested at 24, 48, and 72 hpi and centrifuged to remove cell debris. The media was freeze-thawed three times and then added to Vero cells. After 72 hpi, plaque assay was performed, and the viral titer was determined as described earlier.

### Western blot analysis

PBT- 29FHTC cells were seeded into laminin-coated 60 mm culture dishes at a cell density of 1 × 10^6^. The cells were treated with RSV, hrR3, or either combination. At each time point (24 h, 48 h, and 72 h post-treatment), cells were harvested, and protein isolation was conducted. Protein concentration was analyzed using the Bicinchoninic acid Assay (Pierce BCA Kit #23225 Thermo Scientific, USA). For the western blot analysis, 25 µg of protein resolved in 4–12% Bis-Tri’s sodium dodecyl sulfate-polyacrylamide gel electrophoresis (Bolt Bis-Tris Plus #NW04122, Thermo Scientific, USA). The proteins were transferred to a nitrocellulose membrane using the iBlot2 Gel transfer device. Following the transfer, the proteins were probed with specific primary antibodies obtained from Cell Signaling Technology (Danvers, MA): AKT(#9272S), Phospho-AKT(#9271S), Caspase-9(#9508T), Cleaved PARP (#5625T), Cleaved Caspase-3 (#9664T), STAT3 (#4904T) PSTAT3 (#9145T) and Beta-Actin(#8457S) overnight at 4 °C then membranes were washed with 1x Tris-buffered saline with Tween 20 (TBST) (#R043 Gbiosciences, St Louis, MO). Membranes were probed with secondary anti-rabbit (#711035152, Jackson Immuno Research Laboratories Inc. West Grove, PA) /anti-mouse antibody (#31431, Thermo Fisher Scientific, USA) for 1 h, then washed 3 times with 1XTBST. All the antibodies were diluted according to the manufacturer’s instructions. Membranes were then incubated with chemiluminescence substrate (Super Signal West Pico PLUS Thermo Fisher Scientific, USA), and chemiluminescence signal was captured by iBright CL 1500 (Thermo Fisher Scientific, USA). Quantification and analysis were carried out using the ImageJ software.

### Statistical analysis

All data are presented as mean ± standard error of the mean (SEM) derived from a minimum of three independent experiments. Statistical comparisons among multiple groups were conducted using one-way analysis of variance (ANOVA), followed by Dunnett’s post hoc test for multiple comparisons against control groups. For comparisons between two groups, Student’s t-test was employed. A p-value of ≤ 0.05 was deemed statistically significant. Statistical analyses were performed using GraphPad Prism version 10 (Dotmatics, Boston, MA, USA).

## Results

### DIPG/DMG shows poor prognosis and survival rate

**Fig. 1:**
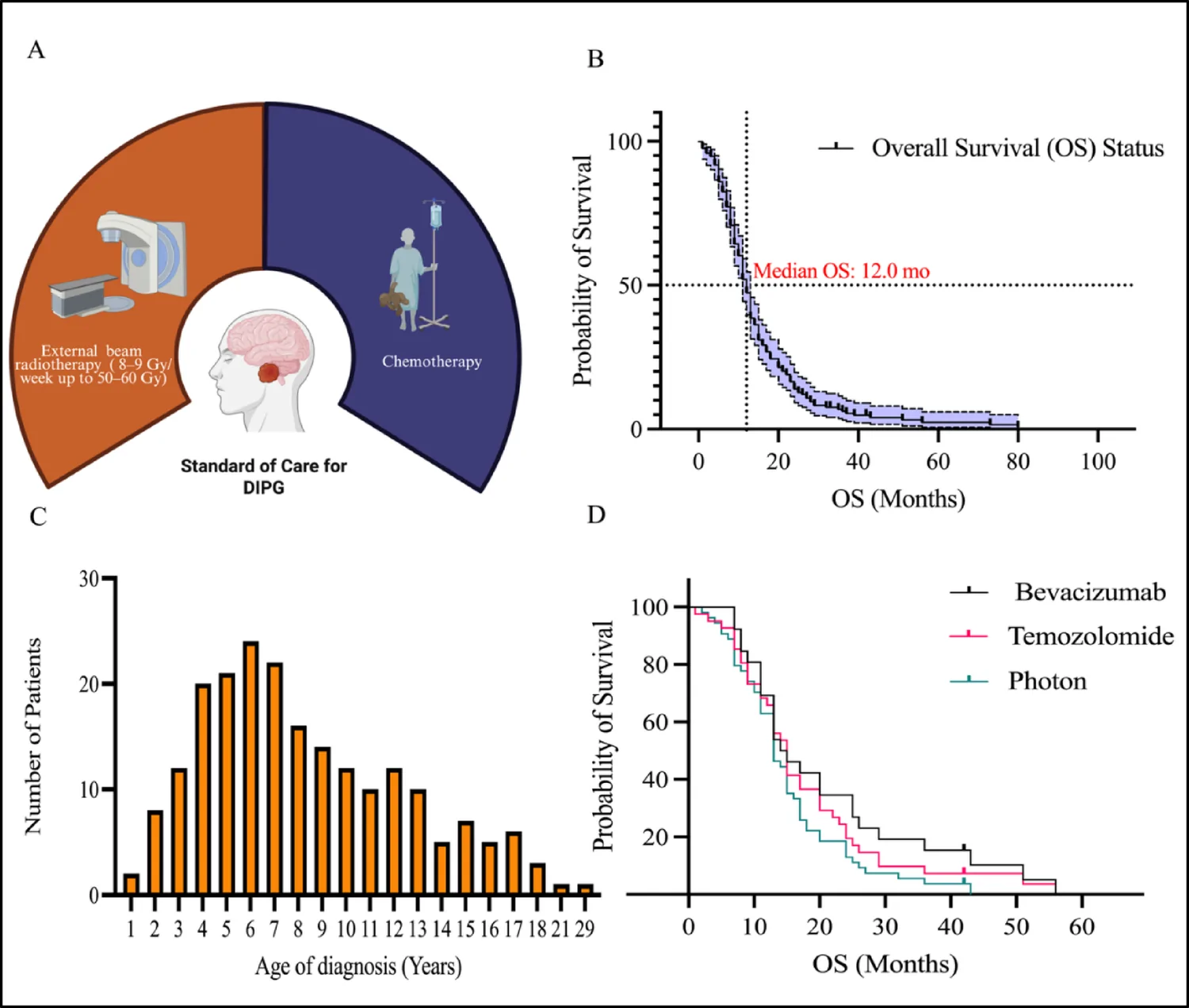
Clinical characteristics and survival analysis of pediatric Diffuse Intrinsic Pontine Glioma (DIPG) patients. **A** A schematic representation of the current standard of care (SOC) for Diffuse Intrinsic Pontine Glioma (DIPG), which primarily includes focal radiotherapy and palliative chemotherapy. **B** A Kaplan–Meier survival curve illustrating the overall survival (OS) of DIPG patients from the time of diagnosis, with dashed lines indicating a median OS of approximately 12 months. **C** An age distribution of DIPG patients at the time of diagnosis, highlighting the number of patients diagnosed at each age and emphasizing the peak incidence in early childhood. **D** Kaplan–Meier survival curves comparing OS among patients treated with bevacizumab, temozolomide, or photon radiotherapy. These data underscore the bleak prognosis for DIPG and the limited survival benefits of currently available treatment options

We initially analyzed clinical data from the PedcBioPortal repository and DIPG remains a devastating pediatric malignancy with a standard of care (SOC) that has not changed in decades. The current SOC relies almost exclusively on a dual approach of external radiotherapy, and palliative chemotherapy (Fig. 1A). The median OS remains anchored at ~ 12 months (Fig. 1B), with a drastic decline in survival within the first two years post-diagnosis. This dismal prognosis is particularly tragic given the demographic most affected by the disease. DIPG is primarily a disease of early childhood, with a peak incidence occurring between 5 and 9 years of age (Fig. 1C). A comparison of contemporary treatment modalities including bevacizumab, temozolomide, and photon radiotherapy reveals nearly identical survival trajectories (Fig. 1D). Collectively, these data provide a quantitative representation of the therapeutic plateau in pediatric neuro-oncology and reinforce the critical need for mechanistically distinct interventions.

### RSV and hrR3 significantly reduce the cell proliferation in DIPG cells

**Fig. 2:**
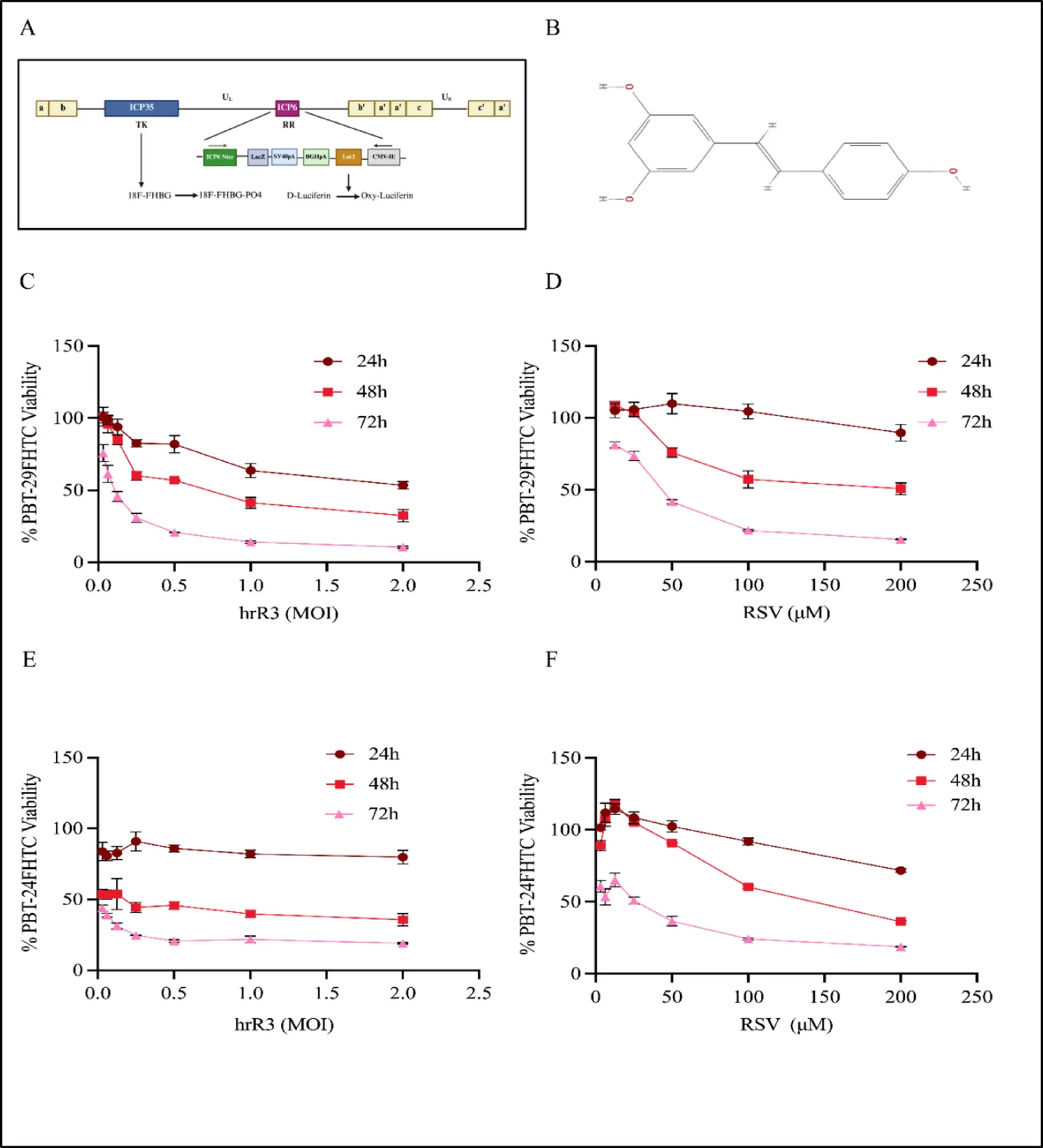
Dose- and time-dependent effects of hrR3 and resveratrol (RSV) monotherapy on diffuse intrinsic pontine glioma (DIPG) cell viability. **A** Schematic representation of the recombinant oncolytic herpes simplex virus hrR3 construct. **B** Chemical structure of RSV. **C**, **E** Cell viability of PBT-29FHTC and PBT-24FHTC cells, respectively, after treatment with hrR3 at the indicated multiplicities of infection (MOIs) for 24, 48, and 72 h post-infection (hpi). **D**, **F** Cell viability of PBT-29FHTC and PBT-24FHTC cells, respectively, after treatment with the indicated concentrations of RSV for 24, 48, and 72 h. Cell viability was measured using the sulforhodamine B (SRB) assay and expressed as a percentage of untreated control cells. Data are presented as mean ± standard error of the mean (SEM) from three independent experiments (*n* = 3). Statistical significance was determined using one-way analysis of variance (ANOVA) followed by post hoc analysis

To evaluate the therapeutic potential of hrR3 (Fig. 2A) and RSV (Fig. 2B) monotherapies, we performed time-course cell viability assays in two patient-derived DIPG cell lines, PBT-29FHTC and PBT-24FHTC. Treatment with hrR3 resulted in a dose- and time-dependent reduction in tumor cell viability at 24, 48, and 72 hpi (Fig. 2C, E). RSV monotreatment led to a concentration-dependent decrease in cell viability in both cell lines (Fig. 2D, F). The IC₅₀ values for RSV were 42 µM for PBT-29FHTC and 150 µM for PBT-24FHTC, indicating differential sensitivity between the two cell lines. For hrR3, an MOI of 0.1 was selected based on the half-maximal inhibitory concentration (IC₅₀) determination for both cell lines. Phase-contrast microscopy demonstrated a progressive reduction in live tumor cell density over time across treatment conditions compared to controls (Figure S1A–D). These results demonstrate that both hrR3 and RSV independently suppress DIPG cell viability in a dose- and time-dependent manner.

### RSV potentiates oncolytic efficacy of hrR3 in DIPG cells

**Fig. 3:**
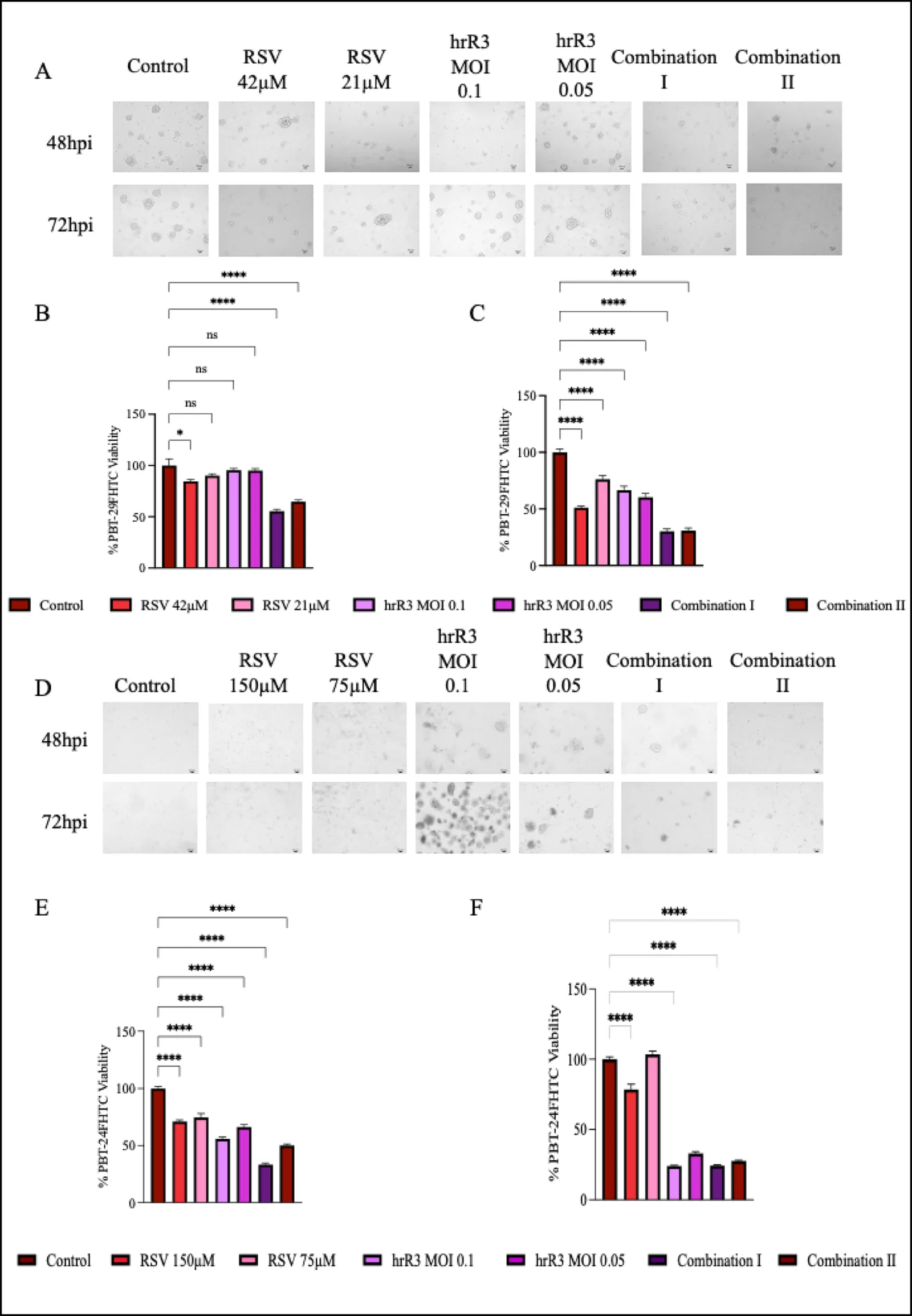
Resveratrol (RSV) enhances hrR3-mediated oncolytic activity in diffuse intrinsic pontineglioma (DIPG) cells. **A**, **D** Representative phase-contrast microscopy images of PBT-29FHTC and PBT-24FHTC cells, respectively, at 48 and 72 h post-infection (hpi) illustrate the morphological changes observed following treatment with RSV alone, hrR3 alone, or a combination of RSV and hrR3 therapy. **B**, **C** Quantitative analysis of cell viability in PBT-29FHTC cells at 48 and 72 hpi under the specified treatment conditions. **E**, **F** Quantitative analysis of cell viability in PBT-24FHTC cells at 48 and 72 hpi in accordance with the designated treatment conditions. Combination I included hrR3 at a multiplicity of infection (MOI) of 0.1 alongside RSV at 42 µM for PBT-29FHTC cells or at 150 µM for PBT-24FHTC cells. Combination II comprised hrR3 at an MOI of 0.05 combined with RSV at 21 µM for PBT-29FHTC cells or at 75 µM for PBT-24FHTC cells. Cell viability was assessed using the sulforhodamine B (SRB) assay and reported as a percentage of untreated control cells. The data are presented as mean ± standard error of the mean (SEM) from three independent experiments (*n* = 3). Statistical analysis was conducted using one-way analysis of variance (ANOVA); ns, not significant; *p < 0.05; ****p < 0.0001 compared with control

**Fig. 4:**
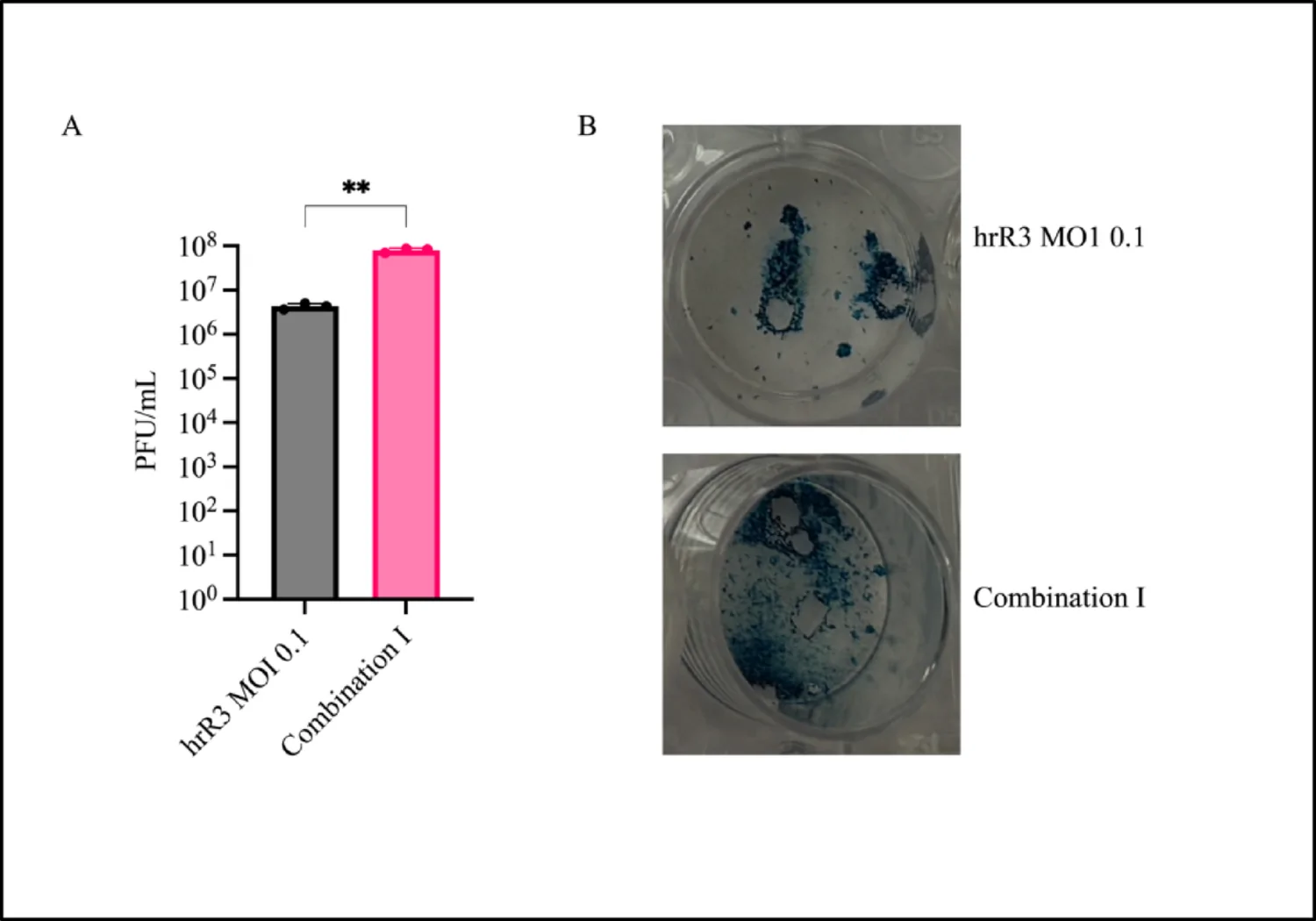
Resveratrol (RSV) enhances hrR3 viral replication in diffuse intrinsic pontine glioma (DIPG) cells. **A** Quantification of hrR3 viral titers from PBT-29FHTC cell culture supernatants collected at 72 h post-infection (hpi). Cells were treated with hrR3 monotherapy at a multiplicity of infection (MOI) of 0.1 or with Combination I treatment consisting of RSV pretreatment followed by hrR3 infection. Viral titers are expressed as plaque-forming units per milliliter (PFU/mL). **B** Representative plaque assay images in Vero cell monolayers corresponding to hrR3 monotherapy and Combination I treatment. Increased plaque formation indicates enhanced production of infectious hrR3 viral particles following RSV pretreatment. Data are presented as mean ± standard error of the mean (SEM) from three independent experiments (*n* = 3). Statistical significance was determined using Student’s t-test; **p < 0.01

To evaluate whether RSV enhances the oncolytic efficacy of hrR3, PBT-29FHTC and PBT-24FHTC cells were treated with RSV, hrR3, or a combination. For ease of understanding the following combinations are being used to detail dosages: Combination I (PBT-29FHTC: hrR3 MOI 0.1 + RSV 42 µM, PBT-24FHTC: hrR3 MOI 0.1 + RSV 150 µM). Combination II (PBT-29FHTC: hrR3 MOI 0.05 + RSV 21 µM, PBT-24FHTC: hrR3 MOI 0.05 + RSV 75 µM). Phase-contrast microscopy revealed that combination treatments led to more extensive cytopathic effects and reduced cell density compared to monotherapy or untreated controls at both 48- and 72 hpi- (Fig. 3A, D). Quantitative cell viability assays further confirmed these observations, with Combination II resulting in a significantly lower viability than either RSV or hrR3 alone (Fig. 3B-C, E-F). Statistical analysis using one-way ANOVA indicated highly significant reduction in viability as compared to controls (****p < 0.0001), suggesting a sensitizing interaction between RSV and hrR3. These findings demonstrate that RSV can potentiate hrR3-mediated oncolysis in pediatric brain tumor models in a dose- and time-dependent manner. To determine the effect of RSV on viral replication, we performed a plaque assay. Results demonstrate that RSV pre-treatment significantly enhanced the viral replication in PBT-29FHTC cells. Combination I formed plaques approximately 8 × 10^7^ plaque-forming units per milliliter (PFU/mL), a significant increase compared to the 4.3 × 10^6^ PFU/mL produced by hrR3 MOI 0.1 alone (**p < 0.001, Fig. 4A-B).

### Suppression of oncogenic STAT3 and AKT signaling in DIPG by RSV and hrR3

**Fig. 5:**
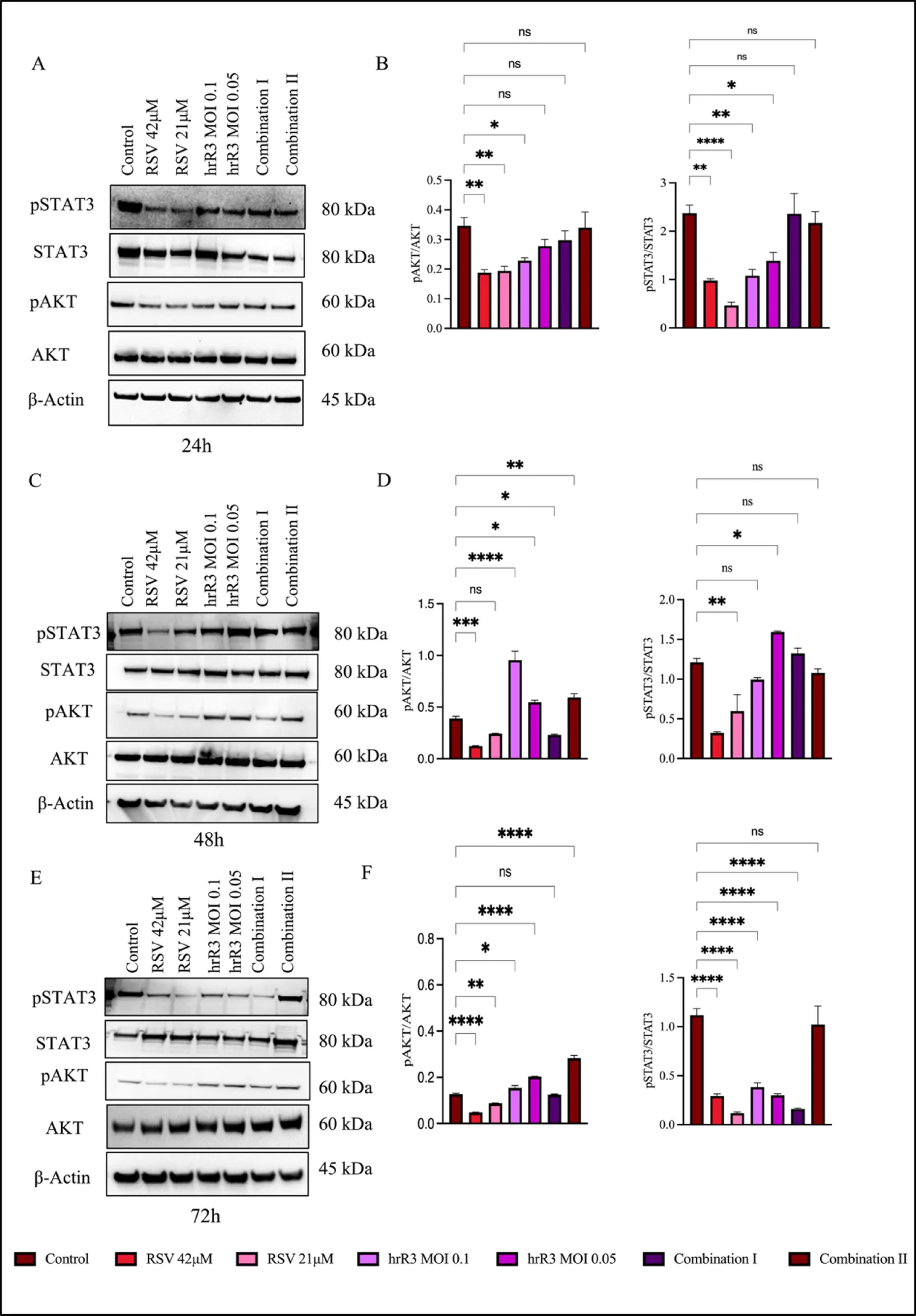
Resveratrol (RSV) and hrR3 modulate signal transducer and activator of transcription 3 (STAT3) and protein kinase B (AKT) signaling pathways in diffuse intrinsic pontine glioma (DIPG) cells. **A**, **C**, **E** Representative western blot analysis of phosphorylated STAT3 (pSTAT3), total STAT3, phosphorylated AKT (pAKT), and total AKT protein expression in PBT-29FHTC cells at 24, 48, and 72 h post-infection (hpi), respectively. The cells were treated with RSV alone, hrR3 alone, or a combination therapy. Combination I included hrR3 at a multiplicity of infection (MOI) of 0.1 along with RSV at 42 µM, while Combination II consisted of hrR3 at an MOI of 0.05 paired with RSV at 21 µM. β-actin served as a loading control. (B, D, F) Densitometric quantification of the pSTAT3/STAT3 and pAKT/AKT ratios corresponding to the western blots depicted in panels A, C, and E. Data are expressed as mean ± standard error of the mean (SEM) derived from three independent experiments (*n* = 3). Statistical significance was assessed using one-way analysis of variance (ANOVA); ns, not significant; *p < 0.05; **p < 0.01; ***p < 0.001; ****p < 0.0001

To assess the impact of RSV and hrR3 treatment on STAT3 signaling, we performed immunoblot analysis to measure phosphorylated STAT3 (pSTAT3) levels in DIPG cells. As shown in Fig. 5, hrR3 monotherapy induced a biphasic increase in pSTAT3 expression, with upregulation observed at 24 hpi (Fig. 5A, quantified in Fig. 5B), peaking at 48 hpi (Fig. 5C, quantified in Fig. 5D), and remaining elevated at 72 hpi (Fig. 5E, quantified in Fig. 5F). RSV pretreatment followed by hrR3 infection significantly reduced pSTAT3 expression compared to both control and hrR3-treated groups. While RSV monotherapy produced a moderate decrease in pSTAT3 levels, the combination treatment resulted in near-complete suppression, particularly at 72 hpi (Fig. 5F).

To further evaluate the effect on survival signaling pathways, we probed for AKT activation. Control DIPG cells exhibited elevated phosphorylation of AKT, whereas combination treatment markedly reduced AKT phosphorylation compared to control and monotherapy groups (Fig. 5A, C, E, and quantified in Figs. 5B, D, F).

Collectively, these results demonstrate that RSV enhances hrR3-mediated inhibition of oncogenic STAT3 and AKT signaling pathways in DIPG cells.

### Combinatorial therapeutic approach significantly enhances apoptosis marker expression in DIPG cells

**Fig. 6:**
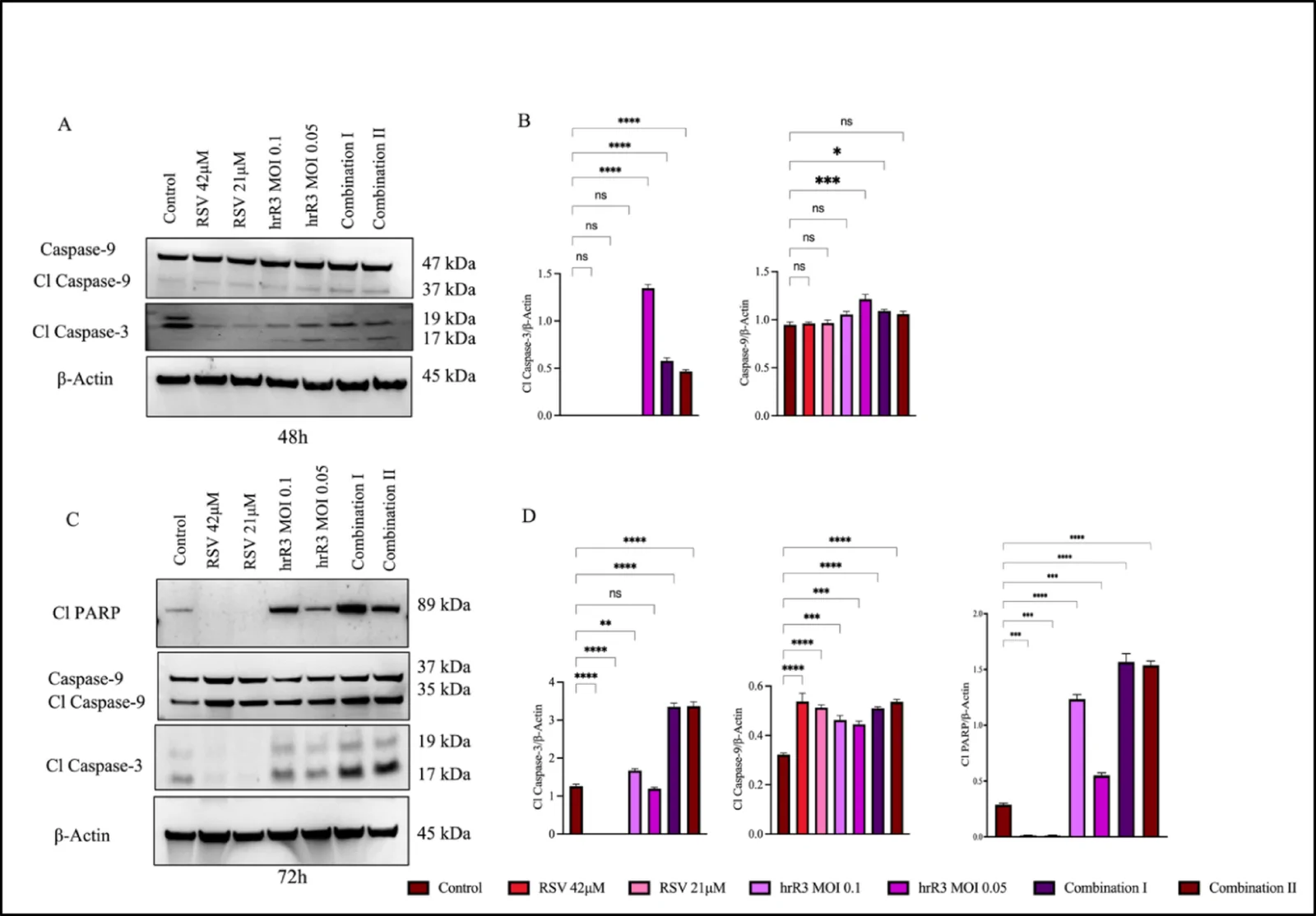
Combination treatment increases apoptotic marker expression in diffuse intrinsic pontine glioma (DIPG) cells. **A**, **C** Representative western blot analysis of apoptotic markers in PBT-29FHTC cells at 48 and 72 h post-infection (hpi). The cells were treated with either RSV alone, hrR3 alone, or a combination therapy. Combination I included hrR3 at a multiplicity of infection (MOI) of 0.1 along with RSV at a concentration of 42 µM, while Combination II comprised hrR3 at an MOI of 0.05 paired with RSV at 21 µM. The apoptotic markers assessed included caspase-9, cleaved caspase-9, cleaved caspase-3, and cleaved poly (ADP-ribose) polymerase (PARP). β-actin served as the loading control. **B**, **D** Densitometric analysis quantified cleaved caspase-3, the cleaved caspase-9/caspase-9 ratio, and cleaved PARP levels normalized to β-actin. Results are presented as mean ± standard error of the mean (SEM) from three independent experiments (*n* = 3). Statistical significance was determined using one-way analysis of variance (ANOVA); ns, not significant; *p < 0.05; ***p < 0.001; ****p < 0.0001

Reduced activation of apoptotic pathways is a hallmark of many cancers, DIPG, contributing to their resistance to therapeutic intervention. To investigate the mechanism of cell death, we analyzed the expression of key apoptotic markers at 48 and 72 hpi via Western blot. In PBT-29FHTC cells, the combination of RSV and hrR3 (Combination I) led to a significant increase in the cleavage of Caspase-3, Caspase-9, and PARP compared to monotherapies at both time points (Fig. 6A, C).

Densitometric quantification revealed that Combination I consistently yielded the highest levels of cleaved Caspase-3 and cleaved PARP relative to β-actin controls (Fig. 6B, D). Specifically, at 72 hpi, Combination I treatment resulted in a dramatic increase in cleaved PARP levels compared to the untreated control and individual therapeutics (*p* < 0.0001). These results indicate that RSV enhances hrR3-mediated oncolysis through the activation of the intrinsic apoptotic pathway.

Additional timepoints (24 hpi) analyzed for temporal progression of apoptosis are included in (Figure S2 A-B) which reveal a time-dependent increase in apoptotic marker activation, culminating in the strongest effects at 72 hpi.

## Discussion

Our findings highlight the therapeutic potential of a combinatorial approach involving RSV and hrR3 in the treatment of DIPG, a highly aggressive pediatric brain tumor with limited treatment options and poor prognosis. DIPG is characterized by resistance to apoptosis, extensive intratumoral heterogeneity, and activation of oncogenic signaling pathways such as STAT3 and phosphoinositide 3-kinase/protein kinase B/mechanistic target of rapamycin (PI3K/AKT/mTOR), which promote tumor proliferation and survival (Nikbakht et al. 2016; Becher et al. 2010; Glaviano et al. 2023). Our findings provide substantial in vitro evidence that RSV significantly enhances the cytotoxicity of hrR3 by reducing the phosphorylation levels of STAT3 and AKT, along with elevating the expression of apoptotic markers. While RSV and hrR3 individually exhibited minimal efficacy on these signaling pathways, their combinatorial effects led to a marked attenuation of survival signaling. This suggests a potentiated therapeutic interaction between RSV and hrR3. A key finding in this study is the enhancement of viral replication associated with RSV pretreatment. Rather than inhibiting hrR3 replication, RSV markedly increased viral titers, suggesting that RSV may alter the intracellular milieu to promote viral propagation. RSV not only impacts viral replication but also modulates various cellular signaling cascades that govern tumor cell survival and stress response mechanisms. Prior research has shown that RSV suppresses the PI3K/AKT pathway and activates p53-mediated signaling, resulting in inhibited cell proliferation and heightened vulnerability to apoptosis (Hedayati et al. 2025; Zhang et al. 2023). RSV has also been reported to activate AMP-activated protein kinase (AMPK) and inhibit mTOR signaling, thereby promoting metabolic stress and enhancing apoptotic and autophagic responses (Zhang et al. 2013). These effects, combined with its ability to modulate redox balance and the nuclear factor kappa B (NF-κB) signaling, may collectively contribute to the sensitization of DIPG cells to hrR3-mediated oncolysis (Leonardi et al. 2021). Our data further demonstrate that the combination treatment is associated with suppression of phosphorylated STAT3 and AKT, both of which are critical regulators of tumor survival and resistance. STAT3 activation has been linked to tumor stemness, immune evasion, and therapeutic resistance in DIPG, particularly in tumors with dysregulated bone morphogenetic protein (BMP) signaling (Furst et al. 2024; Buczkowicz et al. 2014). Similarly, the PI3K/AKT/mTOR pathway, often hyperactivated through receptor tyrosine kinase (RTK) amplifications, drives tumor growth and confers resistance to apoptosis (Mackay et al. 2017). The observed downregulation of pSTAT3 and pAKT in our study indicates that RSV may potentiate the efficacy of hrR3 by inhibiting critical survival signaling pathways. However, without pathway-specific rescue experiments, we must regard these outcomes as correlative rather than establishing direct causality. Accompanying these signaling alterations, we noted a significant elevation in apoptotic markers, such as cleaved caspase-3, caspase-9, and PARP, specifically within the combination treatment cohort. These observations suggest that apoptosis likely contributes, at least partially, to the enhanced cytotoxicity observed. It is crucial to acknowledge that alternative forms of cell death, such as autophagy or necroptosis, might also be involved, though these mechanisms were not investigated in the present study.

### Limitations of the study

All experiments were performed in vitro, which do not adequately mimic the complexities of the tumor microenvironment, particularly immune interactions and BBB dynamics. Moreover, the study utilized only two patient-derived DIPG cell lines, which may not encompass the extensive molecular heterogeneity present among DMG/DIPG subtypes. While the findings included enhanced viral replication and attenuation of STAT3/AKT signaling pathways, direct mechanistic causality through pathway-specific rescue assays was outside the scope of the current study.

### Future directions

Future studies will focus on validating these findings in orthotopic and immunocompetent in vivo models to better recapitulate the tumor microenvironment. Further investigation into antiviral signaling pathways, particularly stimulator of interferon genes (STING) and interferon responses, will be important to elucidate the mechanisms underlying enhanced viral replication. In addition, mechanistic studies involving pathway-specific inhibition or rescue experiments are required to establish causality between STAT3/AKT modulation and enhanced oncolysis. Expanding the analysis to include additional patient-derived DIPG subtypes will improve the generalizability of these findings. Optimization of RSV delivery strategies, including nanoparticle-based or localized approaches, will be critical to overcoming bioavailability limitations. Finally, identification of predictive biomarkers, such as STAT3 or AKT phosphorylation status, may facilitate patient stratification and guide future translational applications.

## Conclusion

In summary, our study suggests that RSV enhances hrR3-associated oncolytic activity in DIPG cells through modulation of survival signaling pathways, induction of apoptotic responses, and potential alteration of antiviral host mechanisms. While these findings are shown in vitro, they provide a strong foundation for further investigation of this combinatorial strategy. This approach may represent a promising direction for improving therapeutic outcomes in DIPG.

## Supplementary Information

Below is the link to the electronic supplementary material.

Supplementary Material 1
