# Supplementary material for: A novel combinatorial therapeutic strategy using resveratrol and viral oncolysis for diffuse intrinsic pontine glioma: an in vitro study
Source: 3 Biotech. 2026 Aug 25;16(9):396. doi: 10.1007/s13205-026-05029-x (PMC13504025; doi:10.1007/s13205-026-05029-x)
Supplement: Supplementary file 1 [file 13205_2026_5029_MOESM1_ESM.docx]

Supplementary figures

**Figure S1. Effect of hrR3 and RSV monotherapy on DIPG cells by phase-contrast microscopy.**

(A, B) Representative phase-contrast images of PBT-29FHTC and PBT-24FHTC DIPG cells treated with varying multiplicities of infection (MOI) of hrR3 over 24–72 hours post-infection (hpi). (C, D) Representative images of PBT-29FHTC and PBT-24FHTC DIPG cells treated with increasing concentrations of RSV over 24–72 hours. Images illustrate dose- and time-dependent changes in cell morphology and viability.

**Figure S2. Temporal analysis of caspase-9 activation at early time point.**

(A) Western blot analysis of total Caspase-9 and cleaved Caspase-9 in PBT-29FHTC diffuse intrinsic pontine glioma (DIPG) cells at 24 hours post-infection (hpi). β-actin was used as a loading control. (B) Densitometric quantification of total Caspase-9 levels normalized to β-actin is shown. Data are presented as mean ± standard error of the mean (SEM) from three independent experiments ($n=3$). Statistical significance was determined using one-way analysis of variance (ANOVA); ns = not significant; **p* < 0.05.

Figure S1:

Figure S2:
